# p300 in Cardiac Development and Accelerated Cardiac Aging

**DOI:** 10.14336/AD.2020.0401

**Published:** 2020-07-23

**Authors:** Asish K Ghosh

**Affiliations:** Feinberg Cardiovascular and Renal Research Institute, Feinberg School of Medicine, Northwestern University, Chicago, Illinois, USA

**Keywords:** heart development, cardiac aging, epigenetics, acetyltransferase p300, extracellular matrix, air-pollutant, hypertension, hypertrophy, fibrosis, heart failure, epidrugs

## Abstract

The heart is the first functional organ that develops during embryonic development. While a heartbeat indicates life, cessation of a heartbeat signals the end of life. Heart disease, due either to congenital defects or to acquired dysfunctions in adulthood, remains the leading cause of death worldwide. Epigenetics plays a key role in both embryonic heart development and heart disease in adults. Stress-induced vascular injury activates pathways involved in pathogenesis of accelerated cardiac aging that includes cellular dysfunction, pathological cardiac hypertrophy, diabetic cardiomyopathy, cardiac matrix remodeling, cardiac dysfunction and heart failure. Acetyltransferase p300 (p300), a major epigenetic regulator, plays a pivotal role in heart development during embryogenesis, as deficiency or abnormal expression of p300 leads to embryonic death at early gestation periods due to deformation of the heart and neural tube. Acetyltransferase p300 controls heart development through histone acetylation-mediated chromatin remodeling and transcriptional regulation of genes required for cardiac development. In adult hearts, p300 is differentially expressed in different chambers and epigenetically controls cardiac gene expression. Deregulation of p300, in response to prohypertrophic and profibrogenic stress signals, is associated with increased recruitment of p300 to several genes including transcription factors, increased acetylation of specific lysines in histones and transcription factors, altered chromatin organization, and increased hypertrophic and fibrogenic gene expression. Cardiac hypertrophy and myocardial fibrogenesis are common pathological manifestations of several stress-induced accelerated cardiac aging-related pathologies, including high blood pressure-induced or environmentally induced cardiac hypertrophy, myocardial infarction, diabetes-induced cardiomyopathy, and heart failure. Numerous studies using cellular and animal models clearly indicate that pharmacologic or genetic normalization of p300 activity has the potential to prevent or halt the progression of cardiac aging pathologies. Based on these preclinical studies, development of safe, non-toxic, small molecule inhibitors/epidrugs targeting p300 is an ideal approach to control accelerated cardiac aging-related deaths worldwide.

## 1. Overview

Heart development is a unique and complex process that occurs very early during embryogenesis [1]. Stress-induced accelerated cardiac aging-related pathologies in adults is the leading cause of deaths worldwide. Improper eating habits, unhealthy foods, sedentary lifestyle, and sleep deprivation-associated increased levels of blood sugar (diabetes), cholesterol (hypercholesterolemia), excessive belly fat (metabolic syndrome), prolonged high blood pressure (hypertension) and disruption in calcium or other electrolyte homeostasis (electrolyte disorder) are the major culprits of accelerated cardiac aging related morbidity and mortality. Numerous drugs targeting signaling pathways and synthetic machinery have been developed to reduce the causative factors of heart disease. However, long-term use of these drugs has severe side effects in a significant proportion of patients. Therefore, development of precision and novel medications with less toxicity is highly desirable for the treatment of heart disease.

As epigenetics plays a significant role in cardio-vascular disease development, epigenetic regulators are ideal targets for therapy. It is worth mentioning that epidrug development is underway for the treatment of variety of cancers [2]. However, very little attention has been paid to developing epidrugs for heart disease. The major epigenetic regulators are acetyltransferases, deacetylases, methylases, demethylases, and microRNAs. This minireview only focuses on the significant role of acetyltransferase p300, a major epigenetic regulator, in heart development and stress-induced accelerated cardiac aging associated pathologies.

## 2. Discovery of Evolutionary Conserved Gene Ep300

The existence of acetyltransferase p300 was originally discovered in the mid-1980s during the search for adenoviral E1A-interacting cellular proteins involved in adenovirus-controlled mammalian cellular growth [3,4]. Using coupled immunoprecipitation/immunoblot analysis of adenovirus-infected mammalian cell lysates and Ad5-E1A antibody, a 250-300 kDa protein, along with four other E1A-interacting polypeptides, was identified. These studies demonstrated that the 250-300 kDa polypeptide formed stable complexes with the E1A proteins and that these complexes controlled mammalian cellular growth and proliferation [3,4]. Furthermore, deletion of N-terminal amino acids 2-36 of E1A prevented the transformation of mammalian cells and correlated with loss of binding to the cellular 300 kDa protein [5]. Therefore, E1A-interacting 300 kDa protein (p300) plays a pivotal role in adenovirus-induced oncogenic transformation and inhibition of mammalian cell differentiation [3-5]. Interestingly, Hasegawa and colleagues [6] reported the role of p300 in cardiomyocyte-specific gene expression where overexpression of E1A in primary rat cardiomyocytes represses α-MHC and β-MHC promoter activity but not β-actin promoter. Importantly, overexpressed mutant p300 which cannot interact with E1A rescues E1A-inhibited β-MHC promoter activity more efficiently compared to overexpressed wildtype p300. These results clearly indicate the pivotal role of physiological level of active p300 in maintenance of cellular homeostasis of differentiated cardiomyocytes [6]. Furthermore, transcriptional complex of p300 or related CBP with bHLH protein MyoD controls cellular differentiation as rat myoblasts treated with antibodies to p300/CBP disrupt functional cooperation with MyoD and abrogates muscle cell differentiation indicating further the significant role of acetyltransferase p300/CBP in cellular differentiation [7].

E1A-interacting p300 gene *(Ep300)* is an evolutionarily conserved gene and has been cloned and characterized in different species including *Drosophila*, *Caenorhabditis elegans,* zebrafish, mice, and humans. In humans, the *Ep300* gene is 87,486 nucleotides [GenBank View: NC_000022.11 (41,092,592.41,180,077)] in size with 31 exons and located on chromosome 22 at q13.2. The molecular characterization of *Ep300 cDNA* [8] revealed that it is 7,245 nucleotides in length and encodes a protein of 2,414 amino acids. The protein has several functional domains—three cysteine/histidine rich (C/H) domains, an acetyltransferase (AT) domain, a bromodomain, and a glutamine rich (Q) C-terminal domain—that interact with numerous transcription factors in a context-dependent manner. Importantly, p300 is a histone acetyltransferase [9] that acetylates histone tails and numerous transcription factors and thus controls target gene expression through chromatin organization as well as activity of the transcription factors (reviewed in [10]).

In *C. elegans,* the p300 homolog cbp-1 is essential for early embryogenesis, as inhibition of *cbp-1* expression leads to developmental arrest of *C. elegans* embryos [11]. *C. elegans* cbp-1 is located on chromosome III with 12 exons. In addition, *cbp-1* loss-of-function leads to shortened lifespan of *C. elegans* [12]. Using a *C. elegans* cbp-1 probe, *Drosophila* CBP (Nejire) was isolated from *Drosophila* libraries. *Drosophila* CBP is located on the X chromosome at 8F-7 with 16 exons. Mutation in CBP causes heart abnormalities in *Drosophila* [13]. In mice, the *Ep300* homolog is located on chromosome 15 [GenBank View: NC_000081.6 (81,585,351.81,652,077)] with 31 exons. *Ep300* deficiency in mice is associated with abnormal heart and neural tube development that leads to embryonic deaths [14]

## 3. Association of Acetyltransferase p300 with Human Diseases

Since the cloning of *Ep300* gene and its molecular characterization, numerous studies have documented the significant physiological role of p300 in maintenance of cellular homeostasis and organismal health through specific interaction with transcription factors, cofactors and signaling molecules of numerous cellular signaling pathways in a context-specific manner. Conversely, dysregulated p300 activity is associated with a wide variety of human diseases. For instance, specific mutations in *Ep300* cause Rubinstein-Taybi Syndrome, which is characterized by mental retardation and craniofacial abnormalities [15-17]. Deletion or translocation of *Ep300* gene and point mutations in *Ep300* gene are associated with a variety of cancers, including leukemia and solid tumors [18,19]. Significantly elevated levels of p300 or its mutant forms are associated with skin, lung, and cardiac fibrosis [20-23]; human failing heart (Ghosh unpublished data); diabetic cardiomyopathy [24]; kidney disease [22,25]; liver disease [26]; metabolic syndrome [27]; senescence and aging [28,29] and long term memory impairment [30]. The homozygous p300 KIX domain point mutation leads to defects in hematopoiesis including anemia, megakaryocytosis and thrombocytosis [31]. This minireview is focused on the role of epigenetic regulator acetyltransferase p300 in heart development and heart disease, with special emphasis on stress-induced accelerated cardiac hypertrophy, cardiomyopathy, matrix remodeling or fibrogenesis, and heart failure.

**Figure 1.**
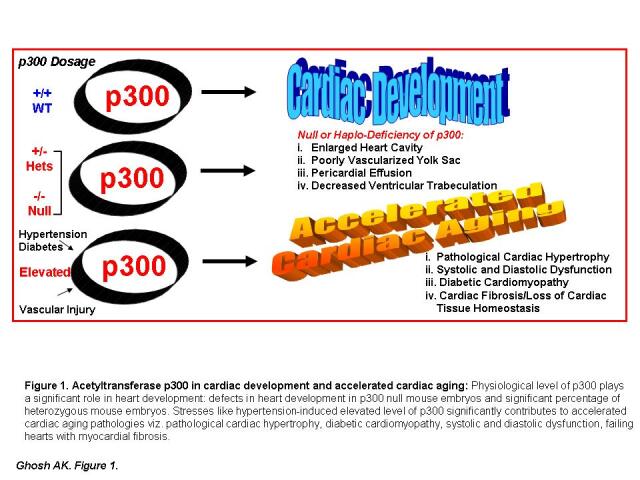
**Acetyltransferase p300 in cardiac development and accelerated cardiac aging**. Physiological level of p300 plays a significant role in heart development: defects in heart development in p300 null mouse embryos and significant percentage of heterozygous mouse embryos. Stresses like hypertension-induced or diabetes-induced or vascular injury-induced elevated level of p300 significantly contributes to accelerated cardiac aging pathologies viz. pathological cardiac hypertrophy, diabetic cardiomyopathy, systolic and diastolic dysfunction, failing hearts with myocardial fibrosis.

## 4. Acetyltransferase p300 in Heart Development

During embryogenesis, murine *Ep300* mRNA is detected at E7.5 [14] before somitogenesis, a series of dynamic processes of segmentation. It is important to note that heartbeats are first detected at 5smites and blood vessel formation at 7somites, which is equivalent to E8 (1-7 somites) [1]. Notably, p300 is essential for mammalian development as mice nullizygous for *Ep300* die during midgestation (E8.5-E11) due to defects in cellular proliferation and neural tube and heart development. Importantly, a significant percentage of *Ep300* heterozygous mice also die in the embryonic stage, suggesting the gene dosage of *Ep300* is an essential factor for cellular growth and differentiation and heart development [14]. Approximately 20% of homozygous *Ep300* mutant embryos show an enlarged heart cavity, a poorly vascularized yolk sac, pericardial effusion, and significantly decreased ventricular trabeculation compared to wild-type control embryos at gestation period E10.5 (see Figure 4 in reference [14]). These features are characteristics of dysfunctional embryonic hearts. The decreased expression of myosin heavy chain and α-actinin in null embryos is associated with weak contractility of cardiac muscle. These results established the pivotal role of physiological levels of p300 in mammalian heart tissue homeostasis.

The contribution of acetyltransferase activity of p300 to mammalian heart development was further evidenced by the fact that mice with a single mutation of p300 lacking acetyltransferase domain leads to embryonic lethality or die within minutes after birth. In contrast only 10% of single allele mutant of CBP lacking acetyltransferase domain are embryonic lethal while rest born alive [32]. The embryonic death of mice with single allele mutation on acetyltransferase domain of p300 are due to defects in heart formation characterized by thin ventricular compact layer, ventricular septum defects (VSD) and underdeveloped atrio-ventricular septum and leaflet (see Fig. 1B a-d in reference [32]). The T-box transcription factor TBX5 plays a pivotal role in cardiac development, and its mutation is associated with congenital heart defects in patients with Holt-Oram syndrome, which is characterized by abnormal skeletal development in the upper limbs and cardiac muscles, especially the ventricular septum, and cardiac conduction-associated bradycardia or atrial fibrillation [33]. Importantly, Murakami and colleagues [34] demonstrated that the WW domain-containing transcription regulator TAZ acts as a TBX5 coactivator and interacts with p300 to stimulate TBX5-dependent expression of genes involved in heart development including atrial natriuretic factor (ANP) and fibroblast growth factor 10 (FGF10). These results signify the significant role of p300 in TAZ/TBX5-dependent cardiac gene transcription and heart development [34].

The significant physiological role of p300 in postnatal cardiac structure and function is supported by the finding that transgenic mice with p300 lacking the cysteine/histidine rich zinc binding domain 3 (C/H3) (p300CH3ΔTG) die by ~20 weeks of age due to cardiomyopathy-associated cardiac dysfunction, including significantly decreased fractional shortening, ejection fraction, left ventricular systolic pressure, and left ventricular end diastolic pressure [35]. Furthermore, Nakagawa *and colleagues* observed deregulated mitochondrial gene expression and membrane potential during the progression of cardiomyopathy in p300CH3ΔTG mice. Their study further implicated down-regulation of PPAR-γ coactivator-1α (PGC-1α), a master regulator of mitochondrial gene expression, in development of cardiomyopathy in p300CH3ΔTG mice. These results strongly suggest the necessity of physiological p300 levels for the maintenance of mitochondrial homeostasis and cardiomyocyte survival in postnatal hearts [35].

Acetyltransferase p300 is also involved with endometrial bleeding-associated factor (EBAF; alternatively, TGF-β4), a NODAL signaling pathway inhibitor [36], during regulation of mammalian heart development. Nodal is known to transduce its signal through Type I and II activin-like receptors and the downstream Smad2/FAST pathway. Interestingly, p300 forms complexes with Smad2 and CBP/p300 interacting transactivator with Glu/Asp rich carboxy-terminal domain 2 (CITED2) and activates EBAF expression through acetylation of histones and chromatin remodeling at the *EBAF* gene promoter. Down-regulation of EBAF in VSD patient tissue is associated with abnormal activation of the NODAL pathway and its target gene pituitary homeobox 2c (PITX2c), which causes ventricular septal defects in humans [37]. Therefore, modulation of p300-EBAF axis to normalize NODAL pathway in VSD patients may improve ventricular septal defects.

An essential role of p300 in mammalian heart development has been further documented by the finding that inhibition of the acetyltransferase activity of p300 with curcumin, a natural polyphenol derived from Asian spice turmeric, during murine development (E11.5) leads to decreased histone acetylation, decreased heart size, thinner ventricular wall and septum, and delayed development of trabeculae and ventricular septum. The expression levels of *Gata4*, *Nkx2.5*, and *Mef2c*, all genes involved in murine cardiac development, were significantly lower in curcumin-treated hearts (E11.5 and E14.5) compared to controls, indicating acetyltransferase activity of p300 is required for cardiac transcription factor expression and maintenance of the normal cardiac development process [38]. Acetyltransferase p300 controls the expression of GATA4 during embryonic mouse cardiogenesis through acetylation of H3K4, H3K9, and H3K27 in GATA4 promoter further indicating the significance of p300 in regulation of transcription factors involved in cardiac development [39]. This has been further supported by the observation that antineoplastic agent doxorubicin-induced cardiotoxicity is due to doxorubicin-promoted selective proteasomal degradation of p300 and rapid depletion of MEF2c and NKX2.5 in cardiomyocytes [40]. Importantly, overexpressed p300 reverses cardiomyocyte gene transcription in the presence of doxorubicin, further indicating that physiological levels of p300 are essential to maintain cardiac homeostasis. It is also important to note that the expression patterns of p300 in the left and right ventricles are different and are correlated with the differential expression patterns of cardiac genes, including atrial natriuretic peptide and brain natriuretic peptide implicating cardiac zone-specific role of p300. The cardiac zone-specific expression patterns of p300 and differential regulation of genes may be due to the different workload in left and right ventricles under physiological and pathophysiological milieus [41]. Together, these studies conclusively establish the essential role of acetyltransferase p300 in embryonic heart development as well as in maintenance of cardiac homeostasis in adults (Fig. 1).

## 5. Acetyltransferase p300 in Cellular Dysfunction and Accelerated Cardiac Aging.

### 5.1. Acetyltransferase p300 in Profibrogenic Signal-induced Accelerated Matrix Remodeling: Lessons from Cellular Models of Tissue Fibrogenesis

Acetyltransferase p300 controls both chromatin organization and transcriptional regulation, the two major regulatory events of eukaryotic gene expression, through acetylation of histones in chromatosomes and transcription factors, and through interacting with transcriptional initiation complex of target genes as a bridging factor between the basal transcriptional machinery and upstream enhancer elements [10]. The very first evidence on the essential role of p300 in profibrogenic cytokine TGF-β-induced synthesis of Type I collagen was reported in 1999 Collagen Gordon Research Conference, and 2000 [42]. Type I collagen is the major contributing extracellular matrix protein in fibrogenesis and hardening of different tissues including heart, lung, kidney and skin [20-26]. We have demonstrated that the acetyltransferase activity of p300 and the interaction of wild-type p300 with phospho-Smad2/3 are essential for elevated matrix protein collagen synthesis and secretion by fibroblasts [42]. Acetyltransferase p300 also plays a pivotal role in the antagonistic action of IFN-γ on TGF-β-induced matrix protein collagen synthesis [43]. Furthermore, wild-type p300, but not acetyltransferase domain-deleted p300 mutant, stimulates the synthesis of Type I collagen (both COL1A1 and COL1A2) and Smad binding element driven reporter gene expression [42,44-46]. Importantly, TGF-β fails to stimulate Type I collagen synthesis in p300 depleted fibroblasts even in the presence of closely related CBP indicating strong link of p300 with elevated matrix protein collagen synthesis (reviewed in [47]). These results suggest that p300 is an essential epigenetic regulator of profibrogenic signal-induced expression of Type I collagen, the major contributing matrix protein in pathological fibrogenesis. Interestingly, the profibrogenic cytokine TGF-β induces p300 expression through activation of the ERK1/2-MAPK-EGR1 pathway and independent of Smad activation. Chromatin immunoprecipitation analysis revealed that profibrogenic signaling induces the recruitment of p300 to the collagen gene promoter, which, in association with increased histone acetylation, augments collagen synthesis. Importantly, the recruitment of p300 and its interaction with the TGF-β-activated Smad2/3/Smad4-Sp1 complex on the collagen promoter are essential for increased synthesis of collagen, the major extracellular matrix protein in fibrotic tissues. Furthermore, TGF-β fails to induce myofibroblast differentiation, the key event during fibrogenesis, from fibroblasts (FMD), endothelial cells (EndMT), and epithelial cells (EMT) in the absence of functional p300 (reviewed in [47]), Ghosh, unpublished data). Previously, we predicted the possible contribution of EndMT derived myofibroblast-like cells in aging-dependent cardiac-selective fibrosis in global PAI-1 deficient old mice because PAI-1 deficient mouse cardiac endothelial cells are more susceptible to EndMT in response to profibrogenic signal [48]. Interestingly, the levels of p300 are significantly elevated in myofibroblast-like cells derived from primary culture of mouse cardiac endothelial cells (EndMT) in response to profibrogenic cytokine TGF-β [49]. The expression levels of several microRNAs were also altered during EndMT compared to control cells [49]. However, the direct link of elevated p300 with altered expression of microRNAs had not been established in these studies. Later, Feng and colleagues [50] showed the presence of increased EndMT in the wildtype diabetic mice hearts but not in the miR-200b overexpressing transgenic diabetic mice hearts. Interestingly, overexpression of miR-200b abrogates diabetes-induced increased expression of p300, a target of miR-200b, along with other EndMT markers/regulators, and cardiac fibrosis, and that is associated with improvement of diabetes-induced cardiac functional and structural abnormalities. These data therefore signifying the role of elevated p300 in diabetes-induced EndMT and cardiac dysfunction [50]. In a series of studies (reviewed in [47], we demonstrated that dissociation of p300 from the transcription initiation complex formed on the collagen gene promoter, using overexpressed adeno-E1A or STAT1α or p53 or PPAR-γ blocks profibrogenic signaling-induced Type I collagen synthesis [47]. Most importantly, small molecule inhibitor-mediated suppression of acetyltransferase activity of p300 in fibroblasts is associated with suppression of TGF-β-induced histone 3 lysine 9 (H3K9) acetylation, myofibroblast differentiation, and matrix protein collagen synthesis [22]. Therefore, peptide-mediated disruption of p300’s interaction with the collagen promoter transcriptional complex or drug-like small molecule mediated inhibition of p300’s acetyltransferase activity may be ideal approaches to control profibrogenic signal-induced FMD, EndMT, EMT and pathological matrix remodeling associated with accelerated aging related numerous diseases.

### 5.2. Acetyltransferase p300 in Accelerated Cardiac Aging: Lessons from Cellular and Animal Models

While deficiency or deregulation of *Ep300* gene is associated with defective cardiogenesis, p300 abnormality in adults is associated with cardiac disease development (Figure 1). p300 is known to control the transcriptional regulation of a variety of genes involved in cardiac hypertrophy. Cardiac hypertrophy can be categorized as physiological hypertrophy and pathological hypertrophy. Physiological hypertrophy occurs during embryonic heart development and as a consequence of regular physical exercise in adults, as evidenced by larger hearts in athletes. In contrast, pathological hypertrophy occurs due to sustained high blood pressure or abnormalities in prohypertrophic signaling or downstream hypertrophic gene expression [51]. Gusterson *et al.* [52] demonstrated that, while inhibition of p300 activity (using dominant negative mutant p300 that lacks CH1 domain) blocks cardiomyocyte hypertrophy induced by phenylephrine, a vasoconstrictor and an inducer of alpha 1 receptors, overexpression of wild-type p300 stimulates cardiomyocyte hypertrophy—clearly indicating the direct role of p300 activity in regulation of pathological cardiac hypertrophy [52]. Furthermore, phenylephrine induces the expression of p300, acetylation of zinc finger transcription factor GATA4, and its binding to the endothelin-1 promoter in cardiomyocytes. Importantly, a dominant-negative mutant of p300 that lacks the GATA4 binding site and the acetyltransferase domain significantly blocks phenylephrine-induced acetylation of GATA4 and its target endothelin 1 gene (ET-1) expression. These alterations in nuclear transcription factor activity are associated with cardiac dilation and systolic dysfunction, further indicating the significant role of p300 activity in pathological cardiac hypertrophy and decompensated heart failure [53]. Acetyltransferase p300-mediated acetylation of GATA4 and its significance in hypertrophy has been further documented by Takaya *et al.* [54], who demonstrated that p300-mediated acetylation of 4 specific lysine residues on GATA4 are essential for the synergistic activation of atrial natriuretic peptide and endothelin-1 promoters by GATA4, and mutations of these residues suppress hypertrophic responses in cardiomyocytes. Interestingly, a class III histone deacetylase, SIRT6, suppresses phenylephrine-induced cardiomyocyte hypertrophy through induction of p300 degradation and inhibition of its downstream target genes [55], further implicating p300 as an essential epigenetic regulator of stress-induced accelerated cardiac aging associated pathological hypertrophy and an ideal druggable target for its treatment.

Acetyltransferase p300 and associated factors are also involved in prenatal alcohol exposure-induced fetal cardiac hypertrophy. The anacardic acid-mediated inhibition of p300/pCAF and histone acetylation down-regulate the expression of hypertrophic murine genes including *Nkx2.5*, *βMHC*, and *CX43* and reverse alcohol-induced cardiac hypertrophy [56]. Recently, the role of p300 in murine models of air-pollutant particulate matter (PM_2.5_)-induced cardiac pathology has also been reported. This study demonstrated that exposure of pregnant mice to PM_2.5_ during the gestation period leads to increased levels of blood glucose, blood lipids, and cardiac hypertrophy in the offspring [57]. Furthermore, PM_2.5_ exposure in the prenatal period increased the levels of transcription factors GATA4 and MEF2c and their downstream hypertrophic targets α-MHC and β-MHC in the offspring hearts. Most importantly, elevated level of p300 was associated with increased binding of p300 to the *GATA4* and *MEF2c* promoters and with increased H3K9 acetylation in hearts of offspring from mice exposed to PM_2.5_ during gestation [57]. Therefore, this study strongly suggests an epigenetic role of p300 in the regulation of transcription factor activities, downstream hypertrophic gene expression, and pathological cardiac hypertrophy induced by *in utero* exposure to environmental pollutants.

**Figure 2.**
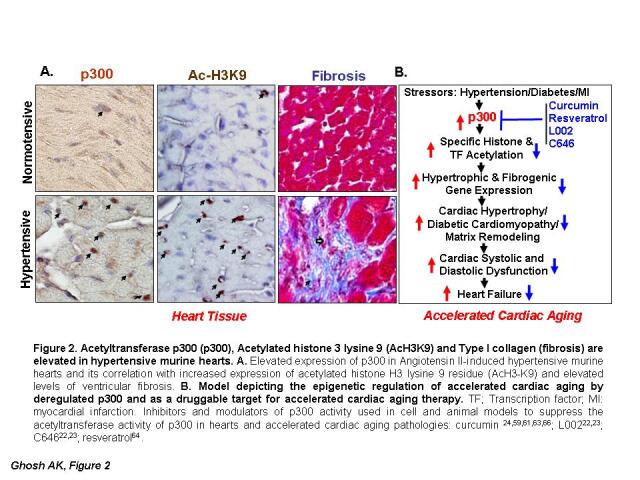
**Acetyltransferase p300 (p300), Acetylated histone 3 lysine 9 (AcH3K9) and Type I collagen (fibrosis) are elevated in hypertensive murine hearts**. **(A)** Elevated expression of p300 in Angiotensin II-induced hypertensive murine hearts and its correlation with increased expression of acetylated histone H3 lysine 9 residue (AcH3K9) and elevated levels of ventricular fibrosis. **(B)** Model depicting the epigenetic regulation of accelerated cardiac aging by deregulated p300 and as a druggable target for accelerated cardiac aging therapy. TF; Transcription factor; MI: myocardial infarction. Inhibitors and modulators of p300 activity used in cell and animal models to suppress the acetyltransferase activity of p300 in hearts and accelerated cardiac aging pathologies: curcumin [24,59,61,63,66]; L002 [22,23]; C646 [22,23]; resveratrol [64,65].

The significance of p300 activity in hypertrophic cardiomyopathy has been further evidenced by the observation that anacardic acid-mediated inhibition of p300/pCAF also ameliorates phenylephrine-induced cardiac hypertrophy [58]. Furthermore, the levels of p300 are significantly elevated in angiotensin (Ang) II-induced hypertensive myocardial tissues and are associated with increased acetylation of H3K9 and cardiac hypertrophy and fibrogenesis (Figure 2A, and also see [22,23]). In a recent report, we have demonstrated that the cell-permeable small molecule L002, a potent and unique inhibitor of p300, prevents hypertension-induced cardiac hypertrophy and cardiac fibrogenesis in a murine model as evidenced by reduction of hypertension-induced heart weight, ventricular wall thickness, and myocardial matrix remodeling or fibrosis [22]. L002 ameliorates hypertension-induced accelerated cardiac aging pathologies through suppression of H3K9 histone acetylation, AT-1 receptor expression, and matrix protein collagen synthesis [22]. Furthermore, treatment of hypertensive mice with L002 or C646, small molecule inhibitors of p300, effectively reverses hypertension-induced left ventricular wall thickening and cardiac fibrosis through suppression of H3K9 acetylation and myofibroblast differentiation [23]. These results are consistent with our original observation in cell culture model that p300 is an essential epigenetic regulator of profibrogenic signal-induced synthesis of collagen [42], the major matrix protein in fibrotic tissues and thus p300 is a potential druggable target for therapy. However, the beneficial effect of p300 inhibitors L002 and C646 on cardiac hypertrophy and pathogenesis of fibrosis is independent of improving AngII-induced high blood pressure. Therefore, combination therapy of small molecule p300 inhibitors and antihypertension drugs will be more effective in ameliorating cardiac pathologies associated with sustained high blood pressures, as indicated by Sunagawa *et al* [59].

The specific role of p300 activity in myocardial infarction (MI)-induced ventricular dilation and cardiac dysfunction has been determined using transgenic mice overexpressing wild-type p300 or acetyltransferase domain mutant p300. Mice overexpressing the wildtype p300 showed left ventricular dilation and reduced cardiac function compared to mutant p300 overexpressing mice or wild-type mice after MI, indicating acetyltransferase activity of p300 is essential for MI-induced accelerated cardiac aging pathologies including hypertrophy and cardiac dysfunction. Overexpressed p300-induced cardiac hypertrophy and cardiac dysfunctions are partly due to p300-mediated hyperacetylation of GATA4, altering its DNA binding activity to downstream activated genes including endothelin-1 and atrial natriuretic peptide [60]. The significance of p300 activity in heart failure has also been evidenced by hypertensive heart disease in salt-sensitive Dahl rats and ligation-induced heart failure models [61]. This study demonstrated that the acetyltransferase inhibitor curcumin, a turmeric-derived polyphenol, inhibits phenylephrine-induced p300/ GATA4 complex formation, GATA4 acetylation, and DNA binding activity of GATA4, a zinc finger transcription factor [61]. Disruption of GATA4-p300 transcriptional complex formation is associated with inhibition of hypertension responsive genes viz. ANP and β-MHC in rat cardiomyocytes. Curcumin mediated inhibition of p300 acetyltransferase activity, decreased GATA4 acetylation and disruption of p300 interaction with GATA4 prevents hypertension- and MI-induced damage of cardiac structure and function as evidenced by improved ejection fraction, fractional shortening, posterior wall thickness, and myocardial fibrosis, without altering blood pressure [61]. The pivotal role of p300 in MI-induced accelerated cardiac aging pathologies has been further documented by Sunagawa *et al.* [59] in a rat model of heart failure. Curcumin treatment improved left ventricular fractional shortening. However, along with ACE inhibitor enalapril, curcumin further improves cardiac performance, indicating that combination therapy with blood pressure-lowering drugs and a p300 inhibitor will be an ideal approach to control heart failure-related accelerated cardiac aging pathologies. Although curcumin is a non-toxic natural product, one of the major limitations of curcumin usage as a drug was its poor bioavailability. To overcome that hindrance, nano-curcumin compound with high bioavailability has been developed and used in preclinical investigations. However, it is still challenging to consider curcumin as an epidrug for accelerated cardiac aging pathologies because of its multifaceted role in controlling numerous signaling pathways including NF-kB, MAPK, Akt/PI3 kinase and programmed cell death pathways [62]. Therefore, further modification of this compound is necessary to eliminate its off-target effects and to get approval for considering it as a potential epidrug for accelerated cardiac aging therapy.

The epigenetic regulator p300 also plays a significant role in *Klf15* deficiency-induced cardiomyopathy and aortopathy. Mice deficient for KLF15, a zinc finger transcription factor, develop cardiomyocyte abnormalities due to increased acetylation capacity of p300 that leads to heart failure and aortic aneurysm [63]. Importantly, the level of KLF15 is significantly decreased in failing human hearts and aneurism tissues, which are also characterized by increased p300-dependent acetylation of p53. Inhibition of p300 activity using curcumin rescued the pathological phenotype in *Klf15*-deficient mice, indicating the significance of p300 in *Klf15* deficiency-induced cardiomyopathy and aortapathy [63].

Resveratrol, a polyphenolic compound found in fruits and nuts, has beneficial effects in cardiovascular disease. The cardioprotective effect of resveratrol stems from induction of deacetylase SIRT1, which suppresses cardiac hypertrophy and fibrogenesis and thus improves cardiac diastolic function in the murine model of Duchenne muscular dystrophy. Mechanistically, resveratrol-induced SIRT1 down-regulates p300 through augmentation of its ubiquitination and proteasomal degradation, further indicating the pivotal role of elevated p300 in pathogenesis of cardiomyopathy, the major cause of death of Duchenne muscular dystrophy patients [64]. Furthermore, resveratrol suppresses cardiac hypertrophy, cardiac dysfunction, and tissue fibrosis in two different models of muscular dystrophy: dystrophin-deficient mdx mice and δ-sarcoglycan-deficient TO-2 hamsters [65] Acetyltransferase p300 also plays a key role in the pathogenesis of diabetic cardiomyopathy. Diabetic cardiomyopathy, in patients with diabetes mellitus, is characterized by myocardial fibrogenesis and initial diastolic and later systolic dysfunction that leads to heart failure. Treatment of streptozotocin-induced diabetic rats with p300 inhibitor curcumin blocks diabetes-induced stimulation of hypertrophic regulators like Angiotensinogen, MEF2A, MEF2C, etc., and improved cardiac performance [66]. The role of p300 in diabetic cardiomyopathy has also been studied in a rat model of diabetic cardiomyopathy, TG m(Ren-2)27 [24]. This study demonstrated that treatment of TG m(Ren-2)27 rats with p300 inhibitor curcumin improves cardiac structure-function and reduces cardiac fibrosis as evidenced by reduced diastolic dysfunction, cardiomyocyte hyper-trophy, and myocardial matrix protein deposition.

Taken together, these studies indicate the pivotal role of p300 in accelerated cardiac aging pathologies including cardiac hypertrophy, myocardial infarction, aortic aneurism, diabetic cardiomyopathy and myocardial matrix remodeling. Suppression of p300 acetyltransferase activity by efficient and safe delivery of the natural molecules or of drug-like synthetic small molecule inhibitors of p300, leads to slowdown or reversal of hypertension- and other stress-induced accelerated cardiac aging pathologies. Therefore, the results of all these preclinical studies using cell and animal models clearly indicate that epigenetic regulator p300 is a druggable therapeutic target for stress-induced accelerated cardiac aging pathologies and thus p300 inhibitors can be considered as anti-aging agent (Figure 2B). In this context, it is worth mentioning that acetyltransferase p300 is also involved in blocking cellular autophagy, a key cellular process for removal of toxicity-causing damaged cellular proteins through autophagosomes [67], and a plant product nordihydroguaiaretic acid (NDGA) increases the life span of mice, *Drosophila and C. elegans* through activation of autophagy (reviewed in [29]). It is important to note that p300 is known to acetylate and decrease the stability of autophagy-related proteins including Atg5, Atg7, Atg12, and LC3 those regulate the formation of autophagososme, and thus p300 is a potent blocker of autophagy [29,67]. Recently, Tezil *and colleagues* [29] investigated the underlying possible anti-aging mechanism of NDGA and demonstrated that upon direct binding to p300, NDGA inhibits its acetyltransferase activity, histone acetylation and increases the levels of Atg5, Atg7, Atg12, and LC3 and induces autophagy, and thus protects cells and organisms from accelerated aging [29].

## 6. Perspective of Applicable Knowledge on p300 in Accelerated Cardiac Aging Pathologies

The essential roles of physiological levels of p300 in cellular homeostasis and heart development during embryogenesis are well documented. Dysregulated p300 activity due to somatic mutations or abnormal expression levels, and disruption of its cell type specific interaction with essential transcription factor in adult tissues are associated with numerous accelerated human diseases. Therefore, genetic or pharmacologic correction of p300 activity is the future goal for treatment of these epigenetic diseases. Since the discovery of the pivotal role of p300 in profibrogenic processes and collagen synthesis using a cellular model of fibrogenesis, numerous studies have demonstrated the significance of p300 in fibrogenesis associated with accelerated aging-related numerous diseases in different organs including heart under pathological stresses using different animal models. In the last twenty years, we have gained concrete knowledge that helps us to understand the molecular regulation of accelerated cardiac aging pathologies by p300 and to build a path towards translational science for accelerated cardiac aging therapy targeting epigenetic regulator p300.

Several natural and synthetic small molecule inhibitors of p300 including curcumin, L002, and C646 or p300 activity modulator resveratrol have been used to prevent or halt accelerated cardiac aging associated pathologies. Although the outcomes of preclinical studies clearly indicate the efficacies of these molecules in inhibition of p300, thus decreasing histone and transcription factor acetylation and expression of hypertrophic and fibrogenic genes in a prohypertrophic and profibrogenic milieus, further long-term studies using larger animal cohorts are required to determine the specificity and toxicity of these compounds. In addition, besides its direct role in chromatin remodeling and transcriptional activation through specific acetylation of histones and transcription factors in target gene regulatory domains, p300 is also involved in crosstalk of numerous cellular signaling pathways to control several cellular and organ functions. Therefore, it is important to investigate the influence of different doses of small molecule inhibitors of acetyltransferase p300 on these pathways in a particular preclinical setup to avoid unwanted side effects [68]. Importantly, discovery of p300 inhibitors with better target specificity, solubility, toxicity profile, and cellular uptake is essential to move from cell culture and animal studies to clinical trials. In conclusion, acetyltransferase p300 is a potential epidrug target for the treatment of accelerated cardiac aging induced by hypertension, diabetes, air-pollutant exposure, and hyperchol-esterolemia-associated myocardial infarction that affect millions of patients worldwide.
